# AI-Assisted Strabismus Diagnosis Using Eye-Tracking and Machine Learning

**DOI:** 10.3390/diagnostics16060910

**Published:** 2026-03-19

**Authors:** Malrey Lee

**Affiliations:** The Future Artificial Intelligence Technology Company, Jeonju 54893, Republic of Korea; mrlee@jbnu.ac.kr; Tel.: +82-10-3611-8004

**Keywords:** strabismus, AI-assisted diagnosis, eye tracking, machine learning, ophthalmic imaging, clinical decision support

## Abstract

**Background:** Strabismus diagnosis via the Alternate Cover Test (ACT) lacks quantitative standardization. This study proposes an AI-assisted framework using eye-tracking and machine learning for objective screening. **Methods:** Gaze coordinates were captured using a 60 Hz infrared eye tracker during ACT. Of the 291 initially screened individuals considered, 50 participants were ultimately included after quality filtering, yielding 335 valid samples. Seven algorithms were evaluated, with the dataset split into 294 training and 41 testing samples. Performance was measured by accuracy, sensitivity, specificity, PPV, and NPV. **Results:** Random Forest showed the best performance, achieving 97.56% accuracy (40/41) on the test set. It demonstrated a sensitivity of 1.00, specificity of 0.95, PPV of 0.95, and NPV of 1.00. The confusion matrix confirmed minimal false negatives, ensuring reliable clinical screening. **Conclusions:** The proposed system provides a robust, objective tool for strabismus diagnosis, standardizing ACT interpretation and reducing clinical bias.

## 1. Introduction

Strabismus is a clinically significant ocular disorder for which early detection and accurate evaluation are crucial to achieving successful treatment outcomes [1,2,3,4]. The Alternate Cover Test (ACT) remains the standard clinical procedure for diagnosing strabismus; however, its diagnostic accuracy is often affected by the examiner’s level of experience and the patient’s level of cooperation, particularly in pediatric cases. These factors may lead to considerable variability in clinical interpretation.

To mitigate these limitations, recent studies have increasingly investigated the use of digital gaze-tracking technologies, web-based automated examination platforms, and artificial intelligence-driven analytical methods as alternative or complementary diagnostic tools [5,6,7]. Advances in AI-based screening have shown that automated models can provide high pooled diagnostic performance, with sensitivity and specificity approaching the upper 90% range across diverse datasets, suggesting strong potential for integration with conventional ophthalmic screening [8]. Moreover, AI systems combined with novel sensing modalities—such as wearable eye-tracking hardware—have demonstrated high accuracy in detecting intermittent strabismus by extracting physiologically meaningful ocular features processed through machine-learning algorithms [9]. Deep-learning methods using facial or ocular images have also been successfully applied to automatic detection and classification of strabismus, achieving robust performance in both binary and multi-class classification tasks [10].

Despite these advancements, several critical limitations remain in the current diagnostic workflow. Specifically, there is (1) a lack of a standardized environment for the reliable acquisition of gaze-tracking data during the ACT; (2) an absence of automated algorithms capable of quantitatively analyzing gaze information for objective strabismus assessment; and (3) limited availability of clinically applicable, real-time, web-based diagnostic systems. Although emerging systems combining virtual reality with AI have been explored for pediatric screening, these approaches have yet to be evaluated in real-time web-based clinical workflows that replicate routine examinations [11].

Traditionally, the gold standard for quantifying strabismus has been the Prism Cover Test (PCT). However, the accuracy of PCT is heavily dependent on the clinician’s expertise and can be subject to intra-observer and inter-observer variability. Recent studies, such as those by Cantó-Cerdán et al., have highlighted the advantages of video-oculography (VOG) systems in providing more objective and reproducible measurements of ocular alignment compared to manual prism-based tests. While VOG offers superior precision, its high cost and the need for specialized hardware often limit its accessibility in primary care or resource-constrained settings [12].

Our proposed system aims to overcome these limitations by implementing an eye-tracking-based Alternate Cover Test (ACT) through a web-based platform. By utilizing deep learning architectures to analyze fixation data, our approach provides an objective screening tool that does not require expensive equipment, thereby lowering the threshold for early strabismus detection and expanding diagnostic accessibility to non-specialists.

To address these challenges, this study proposes a web-based diagnostic framework that acquires gaze-tracking data during the ACT and automatically classifies strabismus using an AI-assisted model. The proposed system is designed to replicate routine clinical procedures while enabling real-time acquisition and analysis of eye-movement patterns. By automating the diagnostic process, the framework aims to reduce subjectivity and examiner-dependent variability inherent in conventional manual assessments.

Therefore, the primary objective of this study is to develop and evaluate a proof-of-concept AI-assisted strabismus screening framework that combines eye-tracking during the ACT with machine-learning analysis. Secondary objectives include (1) investigating the impact of preprocessing and multi-sample acquisition, and (2) implementing a web-based prototype for clinical feasibility.

## 2. Materials and Methods

### 2.1. Study Design and Participants

**Figure 1:**
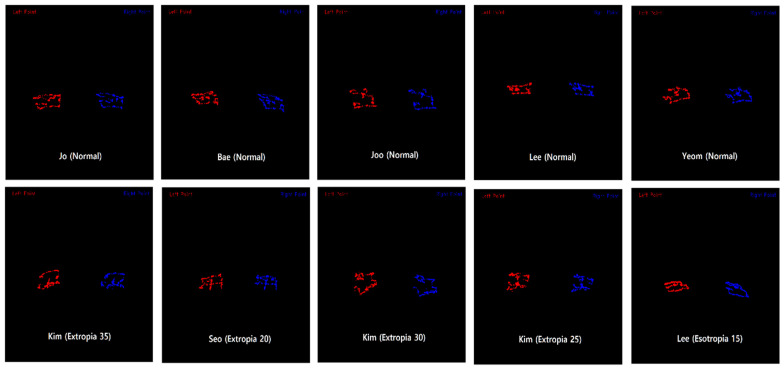
Results of the Nine-Point Experiment Conducted on Actual Patients.

Gaze tracking systems have found successful applications across various industries, demonstrating effective performance in tasks such as object recognition [13], image search [14], and image quality assessment [15]. Furthermore, several previous studies have explored the use of gaze tracking for diagnosing strabismus. Chen et al. introduced a nine-point strabismus test, where patients were instructed to focus on nine points displayed on a monitor for 10 s each, allowing data collection [16]. They utilized 42 sample data points to generate images resembling those depicted in Figure 1 and reported promising outcomes by employing Convolutional Neural Networks (CNN) and Support Vector Machines (SVM) for classification [17]. In real-world scenarios, patients with intermittent or latent strabismus may not exhibit symptoms under ordinary conditions. However, deviations may become apparent when they are instructed to gaze in specific directions. Therefore, prompting patients to look in multiple directions is considered a clinically valuable approach.

Figure 1 illustrates the outcomes of the nine-point experiment performed on real patients. The data in the first row portray the nine-point data from normal patients, while the data in the second row represent strabismus patients, highlighting the direction and angle of strabismus. Despite the variance in format from the image proposed in the original paper, discerning the trend between strabismus and normal patients at a glance is not evident.

The research mentioned above served as significant inspiration for our study. To implement the proposed strabismus diagnosis method in a clinical ophthalmological setting, we acquired two GP3 eye trackers from Gaze Point. Collaborating with an ophthalmology clinic, a total of 291 individuals were initially screened over a three-month period (161 normal and 130 strabismus individuals). Although the environment did not perfectly replicate the conditions proposed in previous papers, reproducing high performance posed challenges due to pediatric patient compliance. Ensuring 100% understanding among pediatric patients was difficult, resulting in noisy data and significant gaze instability.

To ensure data reliability, a strict quality filtering process was applied. Consequently, 50 participants (25 normal, 25 strabismus) were finally included for deep analysis. From these participants, 335 valid diagnostic samples were extracted, where a “sample” is defined as one complete Alternate Cover Test (ACT) cycle from which gaze coordinates were extracted. The final dataset was split into 294 samples for training and 41 samples for testing.

### 2.2. Eye-Tracking Hardware and Protocol

The central mechanism of this study relies on the capability of gaze-tracking systems to monitor pupil movement even when the patient’s eyes are obscured from their view by an infrared-transparent (visible-light-blocking) occluder. Building upon this principle, we conducted the Alternate Cover Test (ACT) using such an occluder to yield clear quantitative distinctions between strabismus and normal cases. A remote infrared GP3 eye tracker (60 Hz, GazePoint, Vancouver, BC, Canada) was utilized for data acquisition. Calibration accuracy, pediatric compliance limitations, and environmental constraints were strictly controlled during the sessions.

**Figure 2:**
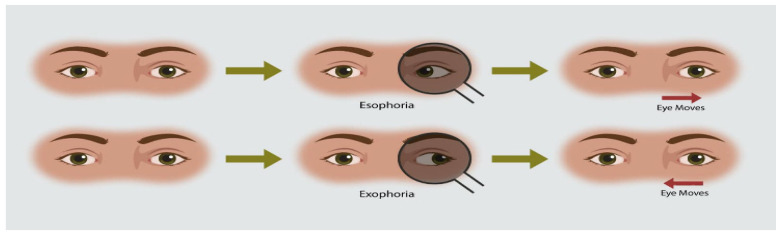
Conceptual Diagram of the Alternate Cover Test (ACT). The ACT entails covering one eye with an infrared-transparent occluder while monitoring the pupil movement of the covered eye. In strabismus, the covered eye deviates according to the strabismus type and returns to the original position upon removal of the barrier.

The protocol for the ACT is illustrated in Figure 2. This classic diagnostic technique involves alternatingly covering the left and right eyes to observe compensatory pupil movement. In strabismus patients, when one eye is covered, the eye behind the occluder shifts in a direction indicative of the strabismus type. Upon uncovering, it returns to the primary position.

As mentioned in Section 2.1, of the 291 individuals initially assessed, 50 participants were finally included after applying predefined inclusion/exclusion criteria. The remaining 241 cases were excluded due to missing frames, incomplete ACT cycles, or insufficient tracking quality (confidence score < 0.25). This rigorous selection ensured a high-quality dataset of 335 valid ACT samples for machine learning modeling.

### 2.3. Dataset Construction

For each included participant, 3–7 ACT samples were obtained, resulting in an initial dataset of 339 samples (106 normal, 4 hypertropia, 188 exotropia, and 41 esotropia samples). Because hypertropia cases (*n* = 4) were insufficient for reliable model training, these samples were excluded, yielding 335 samples for analysis. The dataset was divided into a training set (*n* = 294) and an independent test set (*n* = 41) at the participant level to avoid data leakage (Table 1).

**Figure 3:**
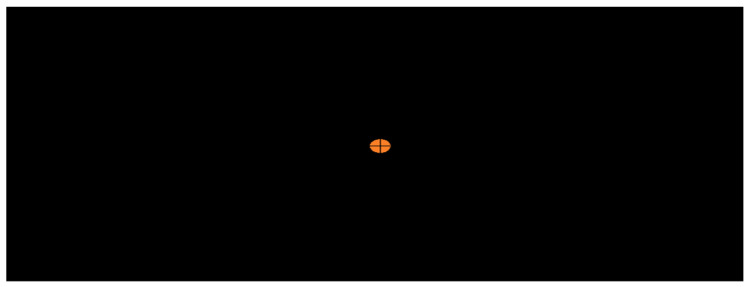
Image Presented to the Patient. The image displayed above is shown to the patient for approximately 10 s. Before the experiment, instruct the patient to focus on the dot for 10 s and inform them that their eyes will be covered with a barrier while maintaining focus.

First, a static image is presented to the patient for approximately 10 s (Figure 3). While the patient focuses on the central dot, the examiner sequentially covers the left and right eyes using an infrared-transparent occluder. The gaze-tracking system reads and digitizes these pupil movements. This observational study used data collected in 2023 at Pureun Ophthalmology Clinic, Jeonju, Republic of Korea, under the supervision of Dr. Sangwon Yoon.

The input data format, including L/RPCX, L/RPCY, and L/RPV, is presented in Table 2. L/RPCX and L/RPCY denote Left/Right Pupil Coordinates X and Y. L/RPV (Left/Right Pupil Valid) takes the value of 1 for valid data and 0 for invalid data. Data points with 0 values were handled during preprocessing to maintain trajectory continuity.

The strabismus group (*n* = 25) included various clinical presentations to ensure diagnostic breadth. The cohort comprised cases of esotropia (*n* = 7) and exotropia (*n* = 18), ranging from intermittent to constant deviations. The severity of ocular deviation was measured using the Prism Cover Test (PCT), with angles ranging from 10 to 45 prism diopters. Participants with a history of prior strabismus surgery or coexisting neurological disorders affecting ocular motility were excluded to maintain the integrity of the initial diagnostic validation.

### 2.4. Preprocessing and Feature Extraction

To enhance the signal-to-noise ratio and improve model robustness, the raw eye data underwent a three-stage preprocessing pipeline: (1) Normalization and Feature Extraction using the difference between right and left eye X-coordinates (RPCX–LPCX); (2) Outlier Removal based on a predefined threshold (0.025) to eliminate tracking artifacts; and (3) Temporal Compression through 10:1 window averaging.

The specific parameters used in the preprocessing pipeline, such as the velocity threshold of 0.025 and the temporal compression ratio of 10:1, were determined through a series of preliminary validation experiments. These values were empirically tuned to achieve an optimal balance between filtering out high-frequency noise from the eye-tracking sensor and preserving the essential saccadic eye movements required for strabismus classification. By testing various ranges, we confirmed that these settings minimize data redundancy without compromising the diagnostic features of the Alternate Cover Test (ACT) sequences.

**Figure 4:**
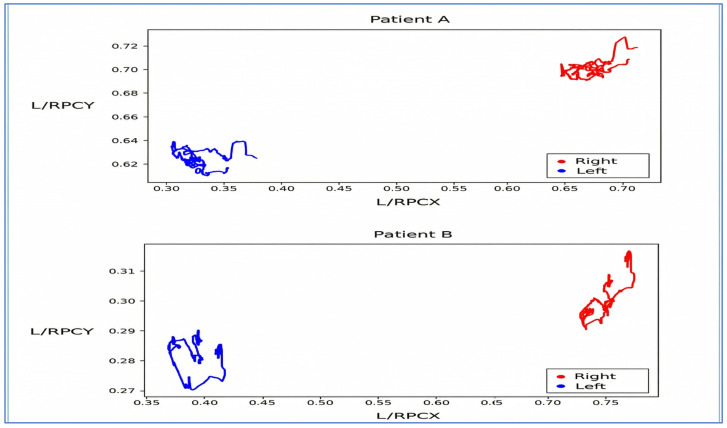
Graph Visualizing L/RPCX and L/RPCY. The graph above illustrates the L/RPCX and L/RPCY data of patients during the Alternate Cover Test.

**Figure 5:**
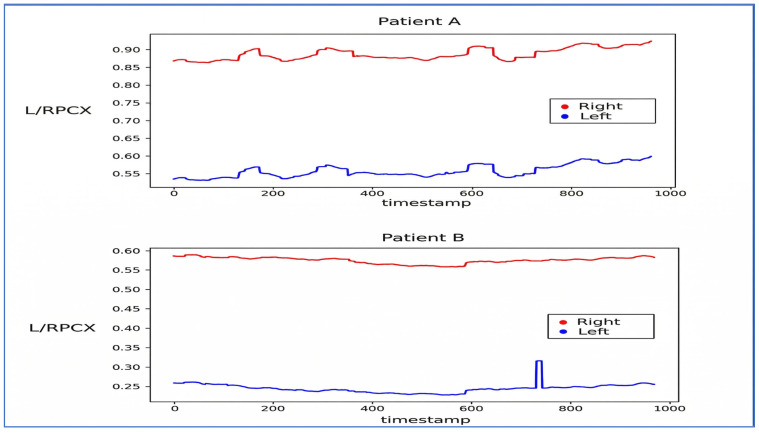
Graph Visualizing L/RPCX Over Time. The graph above illustrates the variation in L/RPCX over time. Patients A and B are unrelated to the data shown in Figure 4.

**Figure 6:**
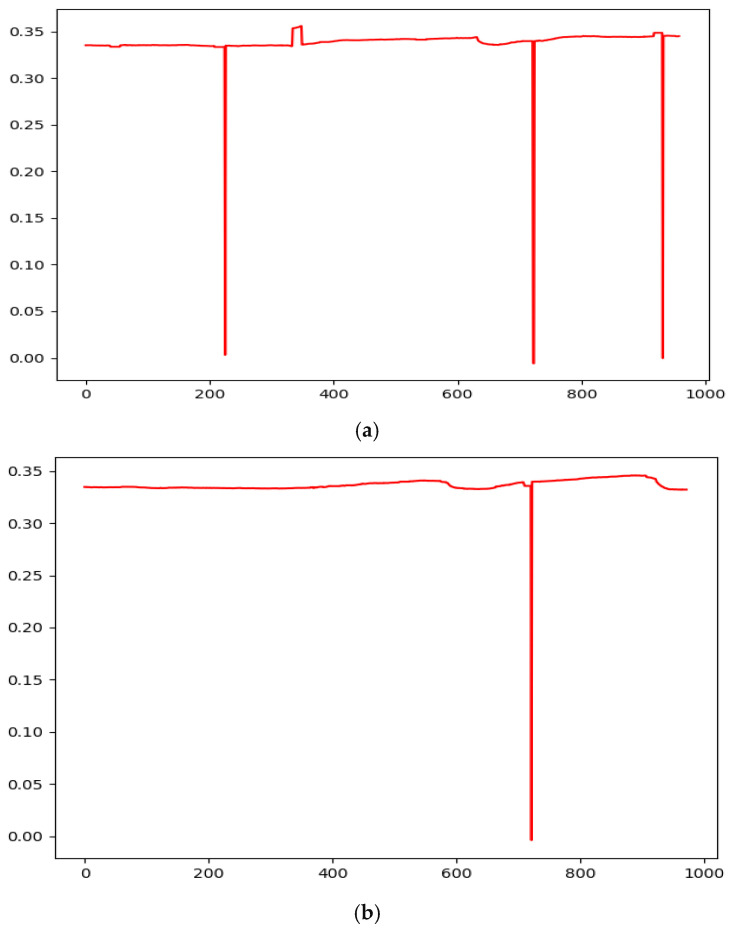
Graph Visualizing RPCX—LPCX Over Time. The graph above illustrates RPCX—LPCX over time, revealing numerous outlier values. Patient A and B are also unrelated to Figure 4 and Figure 5. (**a**) Patient A; (**b**) Patient B.

**Figure 7:**
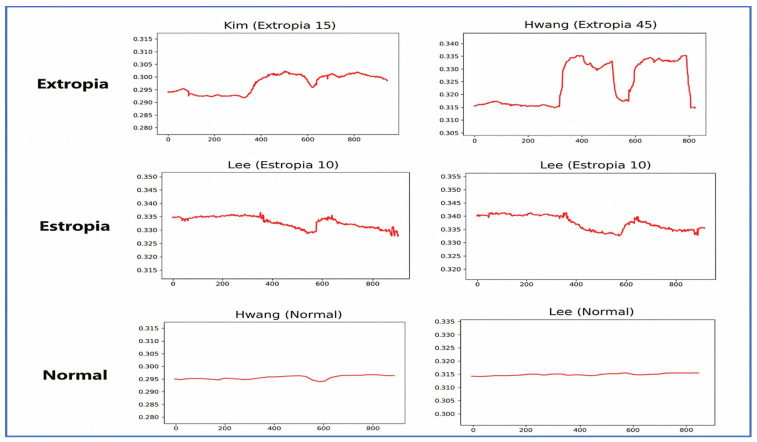
Normalized Eye-Tracking Data.

**Figure 8:**
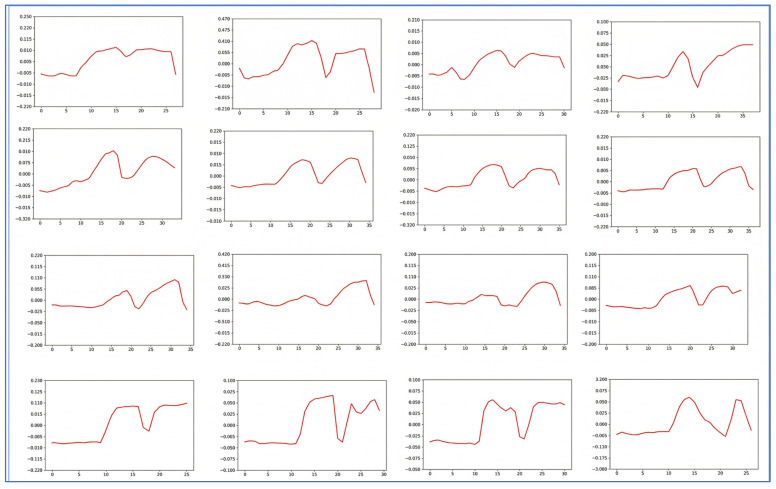
Compressed Strabismic Patient Data.

**Figure 9:**
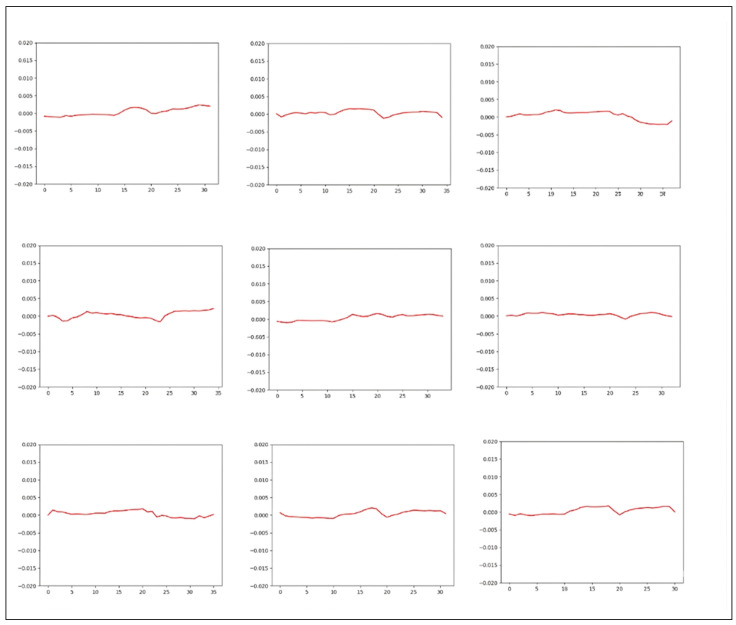
Compressed Normal Patient Data.

Visualization of L/RPCX and L/RPCY was performed to distinguish patterns between strabismic and normal patients (Figure 4 and Figure 5). The L/RPCY feature was removed for *x*-axis dominant movements (exotropia/esotropia), and RPCX—LPCX differences were normalized to remove invalid and outlier data (Figure 6 and Figure 7). Data compression averaged 10 consecutive time steps into 1 time step to simplify patterns (Figure 8 and Figure 9). In all eye-tracking plots, the *x*-axis represents the normalized time steps, and the *y*-axis represents the relative pixel coordinates or coordinate differences.

The key parameters for preprocessing, including the velocity threshold of 0.025 and the temporal compression ratio of 10:1, were determined empirically through preliminary validation experiments. These values were selected to optimize the balance between effective noise reduction and the preservation of essential saccadic and fixation features required for accurate diagnostic modeling.

The graph above illustrates the data after applying a threshold (0.025) to remove time steps where the difference from the average exceeded the set threshold. In this graph, discernible trends between strabismic and normal patients emerge. For exotropic patients, covering one eye leads to an increase in RPCX—LPCX, indicating a divergent deviation typical of exotropia. Conversely, esotropic patients display a decrease in RPCX—LPCX, reflecting convergent deviation. Normal patients exhibit minimal movement, maintaining a stable interpupillary distance.

While Figure 7 effectively distinguishes features between normal and strabismic patients, further simplification is necessary to better instruct the model on the fundamental characteristics of strabismus. For robust classification, the model should capture the underlying patterns of the data rather than being distracted by the excessively fine-grained details present in Figure 7. To address this, we implemented a data compression process by averaging 10 consecutive time steps into a single time step. The resulting compressed gaze-tracking data is presented in Figure 8 and Figure 9.

This figure depicts the compressed eye-tracking data of strabismic patients. By averaging consecutive time steps, the characteristic deviation patterns (distinct peaks and shifts) associated with strabismus become more evident, facilitating more effective feature extraction for the classification model.

This illustrates the compressed data of a normal patient, where no discernible pattern is evident. Figure 8 and Figure 9 represent the compressed data of strabismic patients and normal patients, respectively. As observed in the graphs, a clear distinction between strabismic and normal cases is evident, while most of the subtle features have been eliminated. This type of data was utilized as the final training data for classifying strabismic and normal patients.

### 2.5. Machine-Learning Algorithms and Optimization

To determine the most effective classifier for strabismus screening, seven machine learning algorithms were evaluated: Random Forest (RF), Gradient Boosting (GBM), Support Vector Machine (SVM), k-Nearest Neighbors (k-NN), Multi-Layer Perceptron (MLP), Logistic Regression (LR), and Naïve Bayes (NB).

To enhance diagnostic accuracy and prevent overfitting, we optimized the hyperparameters of each algorithm using a grid search strategy with 5-fold cross-validation on the training set (*n* = 294). For the best-performing Random Forest model, the optimal parameters were determined as: n_estimators = 200, max_depth = 20, min_samples_split = 5, and criterion = ‘gini’.

The ensemble nature of the Random Forest and Gradient Boosting models proved particularly effective in mitigating the impact of remaining noise in the gaze coordinate data. All models were implemented using the scikit-learn library in Python 3.9, and the final evaluation was performed on the independent test set (*n* = 41) that was not used during the training or optimization phases.

### 2.6. Statistical Analysis and Performance Metrics

The diagnostic performance of the machine learning models was evaluated using several key metrics: Accuracy, Sensitivity (Recall), Specificity, Positive Predictive Value (PPV), and Negative Predictive Value (NPV). These metrics were calculated based on the confusion matrix results from the independent test set (*n* = 41). To ensure the reliability of the performance estimates, 95% confidence intervals were considered where applicable. All statistical computations and model evaluations were conducted using Python 3.9 with the Scikit-learn (v1.0.2) and NumPy libraries.

Diagnostic performance was evaluated using accuracy, sensitivity, specificity, and F1-score. To quantify uncertainty, 95% Confidence Intervals (CIs) were calculated for all primary metrics based on the binomial distribution. Furthermore, to assess clinical robustness beyond individual sample-level data, patient-level performance was determined by applying a majority-voting rule to the 3–7 data sequences collected from each participant, ensuring that the final diagnosis reflects the consistency of multiple measurements per subject.

### 2.7. Ethics Statement

This study was conducted in accordance with the ethical principles of the Declaration of Helsinki. Since this research involved the retrospective analysis of fully anonymized gaze-tracking data obtained during routine clinical examinations and posed minimal risk to participants, the requirement for informed consent was waived. The study was determined to be exempt from formal Institutional Review Board (IRB) approval by the Institutional Review Board of JeonBuk National University (JBNU), in accordance with the Korean Bioethics and Safety Act, which allows waivers for research using existing, de-identified clinical data.

## 3. Results

A total of 291 participants were initially screened for eligibility. After strict quality filtering—excluding recordings with low gaze-tracking confidence or excessive blinking—50 participants (25 normal controls and 25 strabismus patients) were finally included. These participants yielded 335 valid ACT samples, which were split into 294 samples for training and 41 samples for testing (88:12 ratio).

### 3.1. Model Performance and Preprocessing Effects

The impact of data preprocessing on model performance is summarized in Table 3. Preprocessing improved the classification accuracy across all seven models by an average of 8.15%. Notably, the Random Forest model’s performance increased by 9.13% after applying noise removal and temporal compression. Furthermore, as shown in Table 4, acquiring multiple samples (3–7) per participant significantly enhanced the stability and accuracy of the models compared to using a single sample, without signs of overfitting.

Table 4 presents a detailed performance comparison, including 95% Confidence Intervals (CIs) to quantify the statistical uncertainty of each model. While the Random Forest model achieved the highest accuracy of 84.38% (95% CI: 73.2–95.6), the Naïve Bayes model performed significantly lower at 57.77% (95% CI: 42.6–72.9). This disparity is primarily due to the high correlation between ocular features in strabismus, which violates the independence assumption of Naïve Bayes. Furthermore, a patient-level analysis was conducted by aggregating the 3–7 samples per participant through a majority-voting rule. This approach effectively filtered out transient tracking errors, resulting in a robust final diagnostic outcome at the individual level.

This approach effectively filtered out transient tracking errors, resulting in a robust final diagnostic outcome at the individual level. Specifically, the majority-voting approach yielded a patient-level accuracy of 97.56%, as detailed in the following section.

### 3.2. Diagnostic Accuracy and Confusion Matrix

Among the evaluated algorithms, the Random Forest model demonstrated superior performance on the independent test set. It achieved an Accuracy of 97.56% (40/41), with a Sensitivity of 1.00, Specificity of 0.95, PPV of 0.95, and NPV of 1.00.

The Confusion Matrix for the Random Forest model on the test set is presented in Table 5. The model correctly identified all 21 strabismus cases in the test set, while misclassifying only one normal case as strabismus.

### 3.3. Clinical Implementation

**Figure 10:**
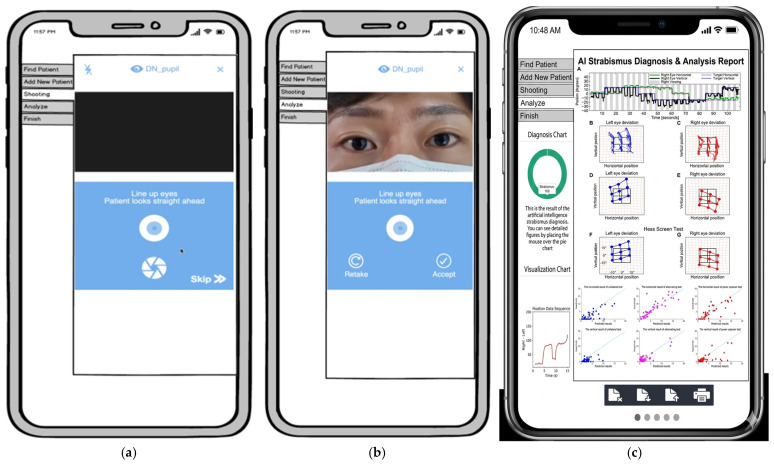
Web-based application GUI for strabismus diagnosis. (**a**) The first screen of the mobile application. (**b**) Capturing the eyes using the application. (**c**) The application displays the diagnostic result, indicating whether the subject has strabismus or is normal.

**Figure 11:**
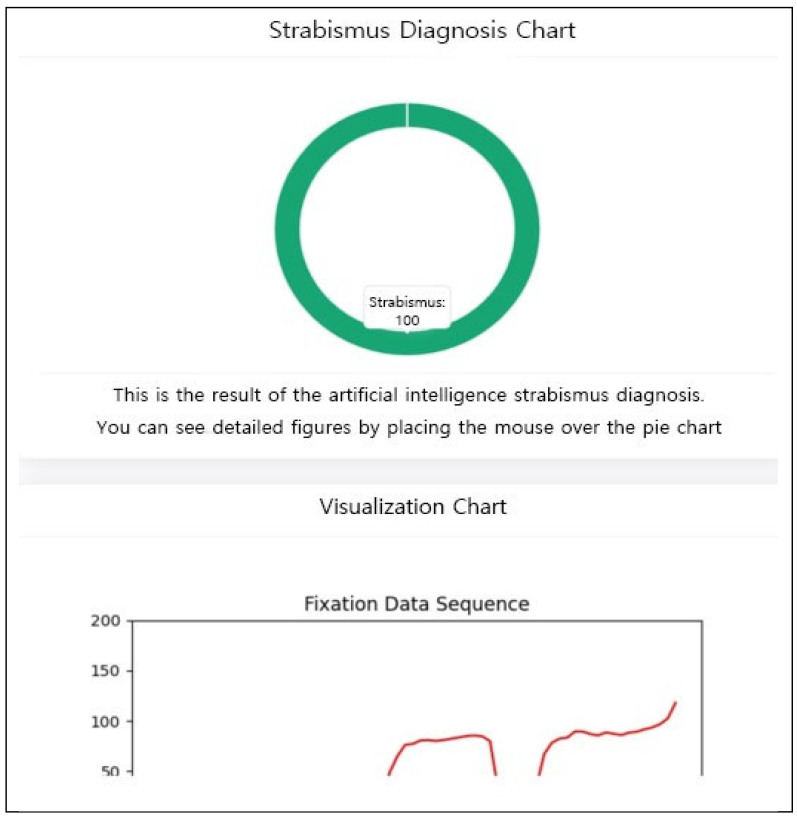
Diagnosis: Strabismus.

**Figure 12:**
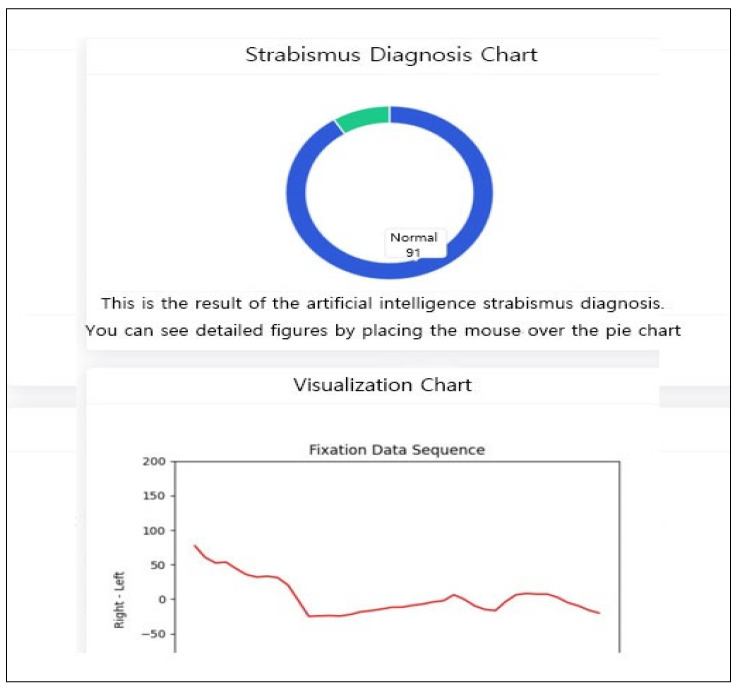
Diagnosis: Normal.

The proposed framework was deployed as a web-based application to demonstrate its clinical feasibility. The system provides real-time visualization of diagnostic results via intuitive GUI components, including probability pie charts and gaze-trajectory line charts (Figure 10, Figure 11 and Figure 12).

## 4. Discussion

The findings of this study indicate that preprocessing strategies and multi-sample data acquisition play a significant role in improving the performance and robustness of AI-assisted strabismus diagnosis. By reducing noise and enhancing the quality of eye-tracking signals, these approaches contributed to more accurate and consistent diagnostic outcomes. Another important contribution of this work is the development of a web-based diagnostic application, which enables standardized strabismus assessment in clinical settings lacking specialized ophthalmologists. This feature addresses a practical limitation of conventional diagnostic workflows and highlights the potential of AI-assisted systems to support clinical decision-making and improve accessibility to ophthalmic care.

Despite these promising results, several limitations should be acknowledged. First, although 291 individuals were initially screened, the final analysis was based on a curated dataset of 50 participants. This high exclusion rate (82.8%), necessitated by strict quality filtering to handle noisy pediatric data and tracking artifacts, may have introduced selection bias and limited the generalizability of the findings. Second, while multiple ACT samples were collected from each participant to enhance model robustness, this approach could increase intra-subject correlation. To mitigate this risk and ensure model independence, we implemented a strict participant-level data split, ensuring that the model was evaluated on samples from individuals completely independent of the training and validation cohorts.

A primary limitation of this study is the high exclusion rate (82.8%) during participant screening, which may affect the external validity and generalizability of the findings to a broader pediatric population. This strict selection was necessary for this initial proof-of-concept study to ensure high-quality data for technical validation. Additionally, while the balanced dataset (1:1 ratio of normal to strabismus cases) allowed for stable model training, it does not reflect the real-world prevalence of strabismus. Future research will involve more diverse, less-filtered cohorts to validate the system’s efficacy in routine clinical practice.

**Figure 13:**
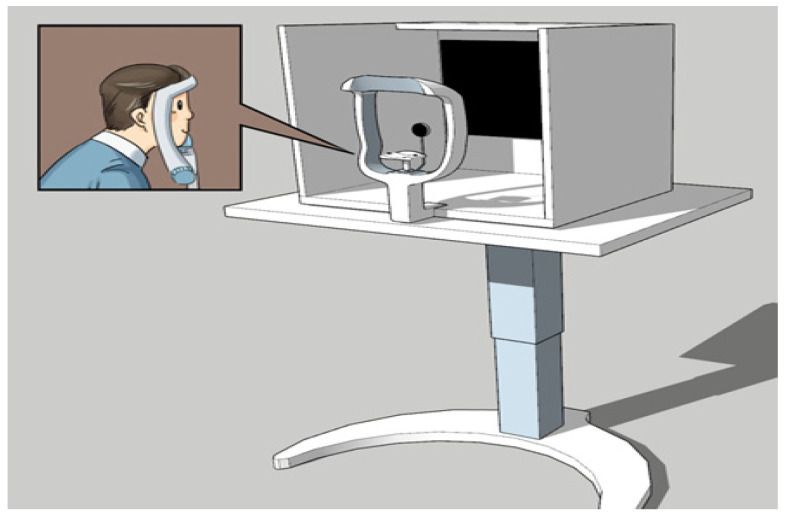
Proposed Improvements for Diagnostic Hardware.

**Figure 14:**
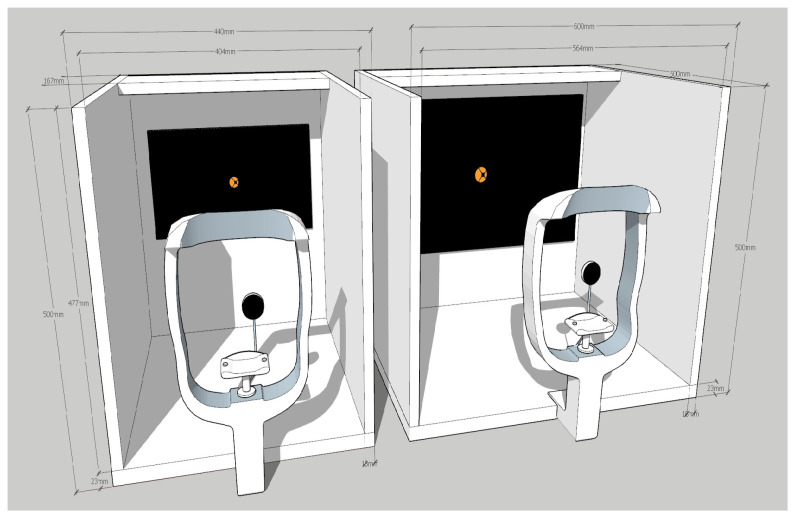
Hardware Optimization for Gaze Stability.

Furthermore, all data were obtained from a single clinical center, which restricts the external validity of our findings across diverse populations and healthcare environments. Technical limitations, such as the lack of physical stabilization and the reliance on manual occluder operation, may have introduced variability in gaze accuracy and measurement consistency. As illustrated in Figure 13 and Figure 14, future iterations of the diagnostic system should incorporate a standardized chin rest to minimize head movement and an automated occluder system to ensure precise and consistent timing during the examination.

Consequently, the results of this study should be interpreted as exploratory, serving primarily as proof-of-concept information rather than definitive clinical evidence. Future work will focus on refining eye-tracking hardware, establishing standardized acquisition protocols, and expanding the dataset through large-scale, multi-center studies involving more diverse patient populations. Such efforts are essential to validate the proposed framework and ensure its robustness prior to real-world clinical deployment. Ultimately, comprehensive multi-center validation is required before this system can be reliably integrated into routine ophthalmic practice.

Current manual procedures require an examiner to cover the eye for 3–6 s, which may introduce temporal variability. Future designs will automate the occlusion timing and standardize the distance between the monitor and the patient to ensure consistent and reproducible measurements.

Figure 14 Hardware Optimization for Gaze Stability. To minimize movement artifacts, future diagnostic equipment should incorporate a chin rest for head stabilization and a centralized fixation target. These enhancements are designed to ensure pupil stability and improve the overall accuracy of gaze-tracking during the examination.

While our web-based application demonstrates potential for deployment in primary care settings or regions with limited access to ophthalmologists, it is important to emphasize that this study is exploratory in nature. Given the preliminary validation and the specific hardware constraints of current eye-tracking technology, the system should currently be viewed as a supportive screening tool rather than a definitive diagnostic replacement. Further large-scale clinical trials are necessary before broad implementation in unsupervised environments.

## 5. Conclusions

This study demonstrates the feasibility and preliminary clinical potential of an AI-assisted strabismus screening framework using gaze-tracking data during the Alternate Cover Test (ACT). By integrating sophisticated preprocessing strategies and multi-sample data acquisition, we achieved a high diagnostic accuracy of 97.56% on an independent test set, significantly improving the robustness of the automated classification.

The proposed web-based application offers a standardized and accessible platform for strabismus assessment, particularly in clinical settings where specialized ophthalmic expertise is limited. By reducing examiner-dependent variability and providing objective, quantitative data, this system addresses a critical gap in current diagnostic workflows. While these results are encouraging, future large-scale, multi-center validation studies are essential to ensure external validity across diverse populations. Continued advancements in eye-tracking hardware and standardized protocols will further enhance diagnostic reliability and facilitate the seamless integration of this technology into routine clinical practice. To ensure the clinical readiness of our web-based platform, future research will incorporate a structured blind study involving independent practitioners to minimize potential observer bias and confirm the system’s efficacy across varied patient cohorts.

## Figures and Tables

**Table 1:** Construction of the final dataset.

Stage	Number	Description
Initially assessed participants	291	161 normal, 130 strabismus
Included participants	50	Passed recording quality criteria
Initial samples	339	3–7 samples per participant
Excluded Hypertropia	−4	Insufficient sample size
Final samples	335	Used for ML analysis
Train set	294	Participant-level split
Test set	41	Participant-level split

**Table 2:** The format of the input data.

Timestamp	LPCX	LPCY	RPCX	RPCY	LPV	RPV
0.00000	0.62102	0.39483	1.00578	0.30799	1	1
0.01619	0.62101	0.39435	1.00578	0.30799	1	1
0.03253	0.6208	0.39385	1.00578	0.30799	0	0

Abbreviations: L/RPCX, L/RPCY = Left/Right Pupil Coordinates X and Y; L/RPV = Left/Right Pupil Valid (1 = valid, 0 = invalid).

**Table 3:** Performance Comparison Before and After Preprocessing Techniques. As evident from the table, applying the proposed preprocessing techniques resulted in improved performance across all models.

Model	Before Preprocessing	After Preprocessing
Random Forest	75.45	84.38 (+9.13)
Gradient Boosting	72.97	83.15 (+10.18)
Support Vector Machine	71.18	79.16 (+7.98)
Nearest Neighbors	66.66	78.94 (+12.28)
Neural Network (MLP)	70.00	71.43 (+1.43)
Logistic Regression	68.26	68.96 (+0.7)
Naïve Bayes	42.42	57.77 (+15.35)

**Table 4:** Comparison of diagnostic accuracy between single-sample and multi-sample data acquisition.

Model	Single Sample Accuracy (%)	Multiple Samples Accuracy (%)	Improvement (%)
Random Forest	81.21 [69.1–93.3]	84.38 [73.2–95.6]	+3.17
Gradient Boosting	71.47 [57.6–85.3]	83.15 [71.7–94.6]	+11.68
Support Vector Machine	75.00 [61.7–88.3]	79.16 [66.7–91.6]	+4.16
Nearest Neighbors	71.42 [57.6–85.2]	78.94 [66.4–91.5]	+7.52
Neural Network (MLP)	68.67 [54.5–82.8]	71.43 [57.6–85.3]	+2.76
Logistic Regression	67.20 [52.8–81.6]	68.96 [54.8–83.1]	+1.76
Naïve Bayes	51.30 [36.0–66.6]	57.77 [42.6–72.9]	+6.47

The table presents a performance comparison between collecting only one data sample per participant and collecting multiple data samples (3–7 samples). The results indicate that multi-sample acquisition consistently improves accuracy and model stability.

**Table 5:** Confusion Matrix of the Random Forest Model (Test Set, *n* = 41).

	Predicted Normal	Predicted Strabismus	Total
Actual Normal	19 (TN)	1 (FP)	20
Actual Strabismus	0 (FN)	21 (TP)	21
Total	19	22	41

TN = True Negative, FP = False Positive, FN = False Negative, TP = True Positive.

## Data Availability

The anonymized coordinate-based eye-tracking dataset and the source code used in this study are publicly available at https://github.com/hyunwoongko/strabismus-recognition (access on 17 March 2026). This dataset does not include any original patient videos or identifiable personal information.
